# Regulation of Sterol Biosynthesis in the Human Fungal Pathogen *Aspergillus fumigatus*: Opportunities for Therapeutic Development

**DOI:** 10.3389/fmicb.2017.00092

**Published:** 2017-02-01

**Authors:** Sourabh Dhingra, Robert A. Cramer

**Affiliations:** Department of Microbiology and Immunology, Geisel School of Medicine at Dartmouth, HanoverNH, USA

**Keywords:** ergosterol, *Aspergillus fumigatus*, triazoles, SREBPs, antifungal agents

## Abstract

Sterols are a major component of eukaryotic cell membranes. For human fungal infections caused by the filamentous fungus *Aspergillus fumigatus*, antifungal drugs that target sterol biosynthesis and/or function remain the standard of care. Yet, an understanding of *A. fumigatus* sterol biosynthesis regulatory mechanisms remains an under developed therapeutic target. The critical role of sterol biosynthesis regulation and its interactions with clinically relevant azole drugs is highlighted by the basic helix loop helix (bHLH) class of transcription factors known as Sterol Regulatory Element Binding Proteins (SREBPs). SREBPs regulate transcription of key ergosterol biosynthesis genes in fungi including *A. fumigatus*. In addition, other emerging regulatory pathways and target genes involved in sterol biosynthesis and drug interactions provide additional opportunities including the unfolded protein response, iron responsive transcriptional networks, and chaperone proteins such as Hsp90. Thus, targeting molecular pathways critical for sterol biosynthesis regulation presents an opportunity to improve therapeutic options for the collection of diseases termed aspergillosis. This mini-review summarizes our current understanding of sterol biosynthesis regulation with a focus on mechanisms of transcriptional regulation by the SREBP family of transcription factors.

## Introduction

*Aspergillus fumigatus* is an environmental filamentous fungus and is the major causal agent of the collection of diseases known as aspergillosis (Kwon-Chung and Sugui, 2013). With increase use of immune suppressive therapies to treat many human diseases, the incidence of invasive aspergillosis (IA) is on the rise with mortality rates between 30–95% (reviewed in Brown et al., 2012). Though comprehensive epidemiology studies are currently lacking for aspergillosis, a recent estimate suggests more than 3 million people have invasive or chronic *A. fumigatus* infection potentially leading to more than 600,000 deaths a year (Gsaller et al., 2016). The major drugs used to treat aspergillosis target ergosterol, the fungal cholesterol equivalent, and belong to the polyene and azole classes of antifungal drugs. Amphotericin B is the major polyene used in the context of IA, however, due to host toxicity concerns it is now primarily used for salvage therapy (reviewed in Gallis et al., 1990; Patterson et al., 2016). The azole class of drugs that target the cytochrome P-450 enzyme eburicol 14α-demethylase (encoded by the cyp51A/B/*erg11A/B* genes) in the ergosterol pathway are the major class of drugs used to treat IA (Patterson et al., 2016). Anti-fungal properties of azoles have been long documented, however, agricultural use of azoles as a fungicide is proposed to lead to azole resistance in *A. fumigatus* (Snelders et al., 2009; Chowdhary et al., 2013). Environmental azole resistant isolates have emerged in clinics throughout the world and are associated with high mortality rates (reviewed in Verweij et al., 2016). Affinity binding analysis reveals azoles directly bind with high affinity to Cyp51 class of proteins in various organisms including *A. fumigatus* (Podust et al., 2001; Warrilow et al., 2010, 2013). In *A. fumigatus*, azoles bind to both Cyp51A and Cyp51B, albeit azole binding is tighter to Cyp51B (Warrilow et al., 2010). It is worth noting that azoles are effective in controlling growth of *Aspergillus*, but they are mostly fungistatic against the majority of *A. fumigatus* isolates (Meletiadis et al., 2007). Thus, a better understanding of the molecular mechanisms associated with sterol biosynthesis is needed to develop new therapeutic strategies particularly in the face of emerging triazole resistance.

## Sterol Biosynthesis in *Aspergillus fumigatus*

**FIGURE 1:**
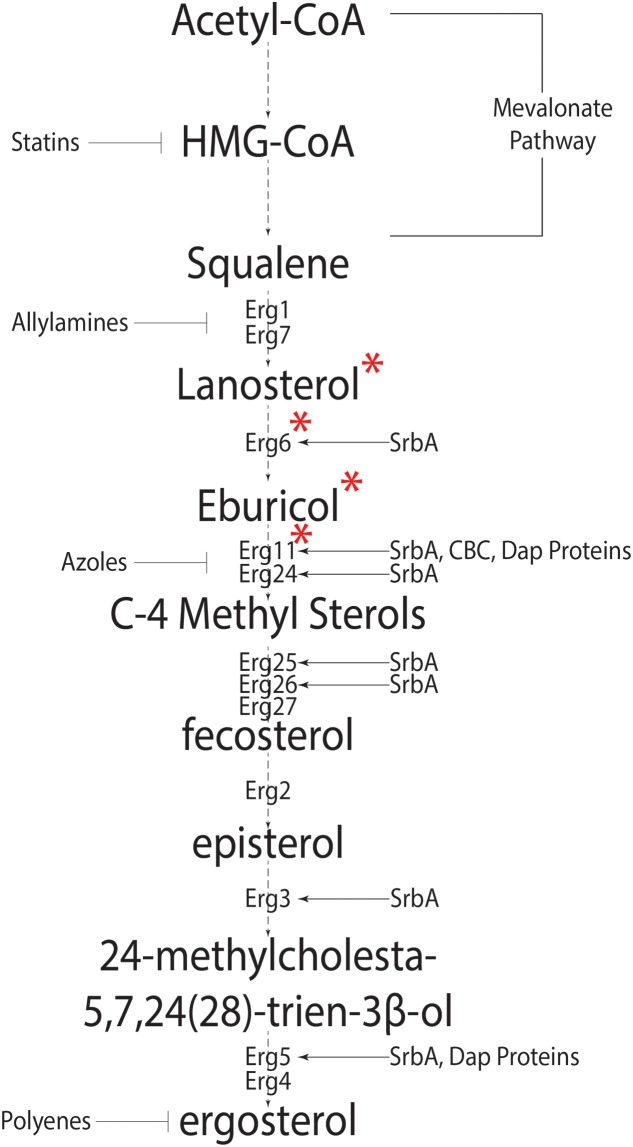
**The canonical fungal ergosterol biosynthetic pathway.** Biosynthesis of ergosterol from Acetyl-coA depicting intermediate steps and enzymes catalyzing the intermediate reactions. Known regulators of Erg genes are shown on right side, whereas anti-fungal agents targeting various pathway steps are shown on the left of the pathway. ^∗^Signifies the pathway differences in *Aspergillus fumigatus* and *Saccharomyces cerevisiae*. In *S. cerevisiae*, Cyp51 converts lanosterol into C4 methylated sterols which are demethylated by Erg24 and Erg25 into zymosterol. Erg6 then converts zymosterol into fecosterol.

Sterols are isoprenoid derived molecules and are a major component of eukaryotic cell membranes; necessary for fluidity, permeability and protein function. Fungi are among the oldest eukaryotes known to synthesize sterols (reviewed in Parks and Casey, 1995). Unlike mammals that have cholesterol as the preferred membrane sterol, fungi synthesize ergosterol. The synthesis of ergosterol begins with acetyl-coA and involves 20 steps (**Figure 1**) (da Silva Ferreira et al., 2005; Alcazar-Fuoli et al., 2008). This process is metabolically costly requiring large amounts of ATP equivalents, reducing power in the form of NADPH, heme-iron, and 12 oxygen molecules (Chang et al., 2007; reviewed in Parks and Casey, 1995; Rosenfeld and Beauvoit, 2003). As molecular oxygen is necessary for sterol production and sterols are only found in eukaryotes, Galea and Brown hypothesize a direct correlation between eukaryotic aerobic life-style and sterols (Galea and Brown, 2009).

Thus, it is plausible that selective pressure exerted by oxygen on primitive life forms led to the emergence of sterols. Oxidative metabolism via mitochondria leads to production of reactive oxygen species (ROS) and eukaryotic organisms have developed strategies to counter intrinsic ROS production (reviewed in Apel and Hirt, 2004; Lambou et al., 2010). Understanding these mechanisms has implications for understanding current sterol targeting antifungal drug mechanisms and in developing new therapeutic approaches. In support of this hypothesis, azole resistant isolates of *C. glabrata* have lower ATP production and lower intrinsic ROS production, possibly due to impairment of mitochondrial function (Peng et al., 2012). In other clinical strains of *C. albicans* and *C. glabrata*, azole resistance shows a strong negative correlation with ROS production (Kobayashi et al., 2002). Thus, oxygen plays an important role in azole mediated sensitivity in various fungi and ROS production plays an integral and yet to be fully defined role in azole drug function. Importantly, given the proposed mechanism of action of the azole class of antifungal drugs on *A. fumigatus*, more research is needed to understand the mechanisms linking sterol biosynthesis, oxygen levels, and fungal fitness during azole therapy.

Along these lines, these observations suggest a role for sterols in preventing oxidative damage by molecular oxygen. In support of a sterol mediated oxidative damage prevention mechanism, exposure of red blood cells (RBCs) to hyperbaric oxygen (HBO), which is associated with oxidative damage, increases RBC’s cholesterol levels (Miliutina et al., 1992). However, the RBC’s oxygen carrying capacity is inversely correlated with cellular cholesterol content (Buchwald et al., 2000). One possible explanation for these observations is that HBO increases oxygen solubility in plasma reducing the load on RBCs to meet cellular oxygen demand. Moreover, 3β-Hydroxysterol-Δ24-reductase (DHCR24), the enzyme that catalyzes the penultimate step in cholesterol biosynthesis, scavenges ROS providing a potential causal link between increased cholesterol levels under prolonged oxygen exposure (Lu et al., 2008). Finally, cats exposed to HBO show a 150% increase in intra-alveolar cholesterol levels (Bergren and Beckman, 1975). However, the relationship between increased sterol levels, ROS resistance, and antifungal drug efficacy remains enigmatic. Some answers may be found in understanding the function and regulation of key sterol biosynthesis enzymes.

Synthesis of HMG-CoA is the first committed step in the biosynthesis of isoprenoids. HMG-CoA reductase catalyzes HMG-CoA to mevalonate and is the rate-limiting in eukaryote sterol biosynthesis (**Figure 1**) (Brown et al., 1973). Two major pathways of ergosterol biosynthesis are proposed after the formation of the first sterol, lanosterol. Lanosterol can be converted to zymosterol or eburicol and this appears to be fungal species dependent. In the model yeast *Saccharomyces cerevisiae* where sterol biosynthesis in fungi is extensively studied, conversion to zymosterol is favored, while eburicol formation is the preferred choice in the human pathogen *A. fumigatus* under conditions examined to date (Fryberg et al., 1973; Nes et al., 1989; Alcazar-Fuoli et al., 2008). Both pathways converge at the formation of fecosterol (Alcazar-Fuoli et al., 2008). Fecosterol conversion to episterol is a unique reversible reaction in the ergosterol pathway; however, evidence suggests that episterol production is favored (Nes et al., 2002). This may explain why fecosterol is not detected in *A. fumigatus* (Alcazar-Fuoli et al., 2008). Three synthetic pathways have been proposed for the conversion of episterol to ergosterol in fungi (reviewed in Nes et al., 1989; Benveniste, 2004). In *A. fumigatus*, intermediates for two pathways have been identified, which suggests at least two of the possible three pathways are functional. **Figure 1** summarizes genes known or predicted to encode enzymes or regulators of the ergosterol biosynthesis pathway in *A. fumigatus.* Importantly, many of these genes remain to be functionally characterized in this important human pathogen.

One potential reason for the lack of genetic analyses on sterol biosynthesis and function in *A. fumigatus* is that many steps in the biosynthetic pathway involve multiple copies of genes encoding the respective enzymes. For example, two 14-α eburicol demethylases (Cyp51A and Cyp51B) (Mellado et al., 2001) and three C5 desaturases (Erg3a, 3b, and 3c) are present in *A*. fumigatus (Alcazar-Fuoli et al., 2006). Mutants lacking *cyp51A* in the ergosterol biosynthetic pathway can grow *in vitro* and are virulent in an IPA murine model (Mellado et al., 2005). This is in contrast with *S. cerevisiae* strains that lack a single *erg11* gene (*cyp51A in A. fumigatus*) and cannot grow aerobically (Bard et al., 1993). However, generation of a double gene replacement mutant (*cyp51A* and *cyp51B*) in *A. fumigatus* is lethal under standard laboratory conditions. Moreover, a strain with *cyp51A* expression under control of a nitrogen source conditional promoter (*niiA)* and a genetic null mutation of *cyp51B* (niiA(p)::*cyp51A*, Δ*cyp51B*) is unable to establish murine infection (Hu et al., 2007). The sterol profile of the *cyp51a* null mutant is similar to WT, however, a complete lack of Cyp51 activity leads to accumulation of 14-α methylated sterols, similar to treatment with triazole antifungal drugs that target this important step in sterol biosynthesis. It is important to note that indigenous host sterol was not able to complement the sterol phenotype of the niiA(p)::*cyp51A*, Δ*cyp51B* double mutant in the IPA murine model (Hu et al., 2007). Thus, it is clear that sterol levels are important for growth and survival of *A. fumigatus* making sterol biology an attractive target for control of aspergillosis.

## Sterol Targeting Drugs Used to Combat Aspergillosis

The fungal membrane maintains cellular homeostasis in part through optimization of phospholipid, sphingolipid and sterol levels. As mentioned, antifungal drugs in the polyene and azole class target cell membrane homeostasis through their effects on sterols. Amphotericin B, a polyene class of anti-fungal drug irreversibly binds to ergosterol and this binding is paramount to fungal killing. Binding of Amphotericin B to sterols in membranes causes membrane leakage and is the proposed mechanism leading to cell death (Gray et al., 2012). A main advantage of Amphotericin B as an anti-*A. fumigatus* drug is its recalcitrance to resistance emergence perhaps due in part to its cidal activity (Meletiadis et al., 2007; Gray et al., 2012). However, its dose dependent toxicity to host cells is a major and significant limitation. Consequently, triazoles (primarily voriconazole and posaconazole) have become the primary choice of treatment for IA (Patterson et al., 2016).

Voriconazole targets heme containing P450 monooxygenase proteins, Cyp51A and Cyp51B, which catalyzes P450 dependent demethylation at C-14 position (reviewed in Ghannoum and Rice, 1999; Mast et al., 2010; Warrilow et al., 2010). Cell membrane integrity requires that embedded sterols lack C-4 methyl groups (Nes et al., 1993). Treatment with voriconazole leads to depletion of ergosterol and accumulation of lanosterol and toxic 14-α-methylated sterols in the plasma membrane. In voriconazole treated *C. albicans*, accumulation of squalene, zymosterol, 4,14-Dimethylzymosterol, 24-methylenedihydrolanosterol and lanosterol is observed (Sanati et al., 1997). In contrast, eburicol and 4α-methyl sterol accumulation is observed in voriconazole treated *A. fumigatus* (Xiong et al., 2005). It is not clear if voriconazole targets additional enzymes in the ergosterol biosynthetic pathway or if these intermediate accumulations are due to indirect effects of accumulating methylated sterols. Voriconazole is effective against itraconazole resistant *A. fumigatus* isolates (Abraham et al., 1999; Dannaoui et al., 2006). It is interesting to note that both azoles target Cyp51 proteins, thus the mechanisms conferring lack of cross resistance needs further investigation (reviewed in Mayr and Lass-Flörl, 2011; reviewed in Odds et al., 2003). Consequently, the azole and polyene drug classes further demonstrate the critical importance for sterol homeostasis in *A. fumigatus* and other fungi.

## Regulation of Sterol Biosynthesis in *A. fumigatus*

### Sterol Regulatory Element Binding Proteins (SREBP) Transcriptional Regulation

Given the evolutionary conservation of sterol biosynthesis in eukaryotes, much can be learned about sterol biosynthesis mechanisms in *A. fumigatus* through examination of more well studied systems in yeast and mammals among others. While many mechanisms are conserved, it is clear that each organism has evolved unique regulatory mechanisms and this is particularly true for *A. fumigatus* as discussed below. Importantly, these unique mechanisms present opportunities to develop novel therapeutic strategies to augment existing antifungal drugs or identify new targets and molecules with activity against *A. fumigatus.*

*De novo* synthesis and LDL receptor mediated endocytosis are two major pathways through which mammalian cells fulfill their sterol requirement (Brown and Goldstein, 1986; reviewed in Espenshade and Hughes, 2007). In mammals, levels of cholesterol are known to control both of these pathways at the transcriptional level (Brown and Goldstein, 1997). The Sterol Regulatory Element Binding Protein (SREBP) class of transcription factors (TF) bind SRE elements and function as major regulators of sterol levels in mammalian cells (Yokoyama et al., 1993; Brown and Goldstein, 1997). When cells are depleted of sterol, the transcription of HMG-CoA reductase and LDL receptor increase through SREBP binding of sterol regulatory element (SRE) DNA elements in promoter regions of these genes. Three major SREBP’s have been identified in mammalian cells viz., SREBP-1a and SREBP-1c encoded by the same gene, and SREBP-2 (Yokoyama et al., 1993; Hua et al., 1995).

Sterol Regulatory Element Binding Proteins are bHLH TFs synthesized as ER resident proteins with their N and C terminals in the cytosol. The SREBP binding partner, SCAP (Sterol Cleavage Activating Protein), regulates the activation of SREBPs (Rawson et al., 1999). When cellular sterol levels are replete, SCAP binds sterols and consequently promotes binding to INSIG (another ER resident protein) to prevent ER exit of SREBP-SCAP. When cellular sterol levels are reduced, SCAP does not bind INSIG which allows ER exit of SREBP via COPII vesicles (Brown et al., 2002; Yang et al., 2002; Sun et al., 2005). In the Golgi, SREBPs are sequentially proteolytically cleaved by golgi resident site-1 serine protease (S1P) that cuts SREBP in the luminal loop (Duncan et al., 1997) and site-2 zinc metallo protease, (S2P) that cuts in the transmembrane domain (Rawson et al., 1997; Duncan et al., 1998). These proteolytic cleavage events release the N terminal TF for nuclear localization and activation of gene expression (Espenshade, 2006; Espenshade and Hughes, 2007).

In the fission yeast *Schizosaccharomyces pombe*, seminal studies identified and characterized fungal homologs of SREBP (Sre1), Scap (Scp1), and Insig (Ins1) (Hughes et al., 2005). As in mammalian cells, Sre1 physically interacts with Scp1 and Sre1 activity is essential to maintain cellular sterol levels through transcriptional regulation of target genes including oxygen dependent steps in the ergosterol biosynthesis pathway (e.g., *erg11, erg25, erg3, erg5, erg6*) (Hughes et al., 2005, 2007a; Todd et al., 2006). A major activation signal for *S. pombe* Sre1 proteolytic cleavage is hypoxia. Under low oxygen concentrations that induce hypoxia in fission yeast, ergosterol levels are reduced and this in turn induces proteolytic cleavage of Sre1. Thus, in fission yeast ergosterol levels act as an indirect oxygen sensor (Hughes et al., 2005). These data further highlight the mechanistic relationship between sterol biosynthesis and oxygen discussed previously. Cleavage of Sre1 is also stimulated by drugs that inhibit ergosterol biosynthesis including the azoles (Hughes et al., 2005, 2007a). Consequently, in fission yeast, ergosterol regulates Sre1-Scp1 ER-Golgi transport, cleavage and activation (Porter et al., 2010). Like mammalian cells, direct binding of ergosterol to Sre1-Scp1 complex determines the fate of Sre1 cleavage, however, unlike mammalian cells, this is independent of Ins1 binding (Porter et al., 2010). In *S. pombe*, the INSIG homolog Ins1 does not regulate the SREBP pathway. Rather, Ins1 regulates HMG-CoA-reductase (*hmg1*) that catalyzes HMG-CoA to mevalonate; the first committed step in sterol synthesis. Unlike in mammalian cells, *S. pombe* Ins1 regulates the activity and not stability of Hmg1 (Burg et al., 2008).

As in fission yeast, *A. fumigatus* contains a membrane bound SREBP homolog, SrbA, required for sterol biosynthesis, hypoxia fitness, iron homeostasis, and azole drug tolerance/resistance (Willger et al., 2008; Blatzer et al., 2011; Chung et al., 2014). The sterol profile of Δ*srbA* shows a significant decrease in total ergosterol content and accumulation of 4-methyl fecosterol and 4,4 dimethyl fecosterol (Willger et al., 2008; Blosser and Cramer, 2012). This likely stems from direct transcriptional control of *cyp51A/B* and *erg25A/B* expression by SrbA in *A. fumigatus* though DNA binding of an SRE motif in their promoters (Blosser and Cramer, 2012; Chung et al., 2014). Overexpression of *cyp51a* in the Δ*srbA* strain does not restore a WT sterol profile or hypoxia fitness which suggests SrbA regulates multiple steps in the ergosterol biosynthesis pathway (**Figure 1**) (Blosser and Cramer, 2012). In fact, over-expression of *cyp51A* in Δ*srbA* exacerbates the accumulation of C4-methyl sterols. However, intriguingly, this strain’s voriconazole and fluconazole MICs are restored to WT levels perhaps due to a decrease in toxic 14-α methylated sterol accumulation (Blosser and Cramer, 2012).

While loss of *cyp51a* or *erg25* is dispensable for virulence of *A. fumigatus*, loss of SrbA severely attenuates *A. fumigatus* virulence in multiple immune compromised IPA murine models (Mellado et al., 2005; Alcazar-Fuoli et al., 2006; Blosser and Cramer, 2012; Willger et al., 2008, 2012). Supporting a partial role for accumulation of C4 methyl sterols as a mechanism to explain the hypoxia fitness defect of Δ*srbA*, generation of an *erg25A/B* double genetic null mutant was not possible in *A. fumigatus* (Blosser et al., 2014). It is thus possible that accumulation of 4-methyl sterols in the absence of SrbA contributes in part to its inability to grow in hypoxia and cause invasive disease. It would be intriguing to examine the sterol profile of Δ*srbA* in the presence of high iron levels where a small but significant restoration of hypoxia growth occurs (Blatzer et al., 2011). Further studies are needed to fully define SrbA’s role in *A. fumigatus* hypoxia fitness and virulence.

Intriguingly and worth consideration for therapeutic development, it is clear that significant differences exist in SREBP regulation between mammals, the yeast *S. pombe*, and the human pathogen *A. fumigatus*. The difference in SREBP activation mechanisms in *A. fumigatus* is highlighted by the observation that a homolog of SCAP is absent in the *A. fumigatus* genome. Moreover, significant levels of the N terminal bHLH portion of SrbA are detectable in normoxia conditions in *A. fumigatus* suggesting that proteolytic cleavage occurs at some level when sterols levels are presumably high. It is worth noting that under these conditions NGFP::SrbA localizes to the ER/NE rather than nuclei (Willger et al., 2012). It is plausible that post-translational mechanisms control the levels of active SrbA, in a manner similar to *S. pombe* (Hughes and Espenshade, 2008), however, this hypothesis needs further investigation. These observations raise an important question that remains to be answered in *A. fumigatus:* how does the *A. fumigatus* SREBP pathway monitor cellular sterol levels? It is plausible that a SCAP like protein remains to be identified. A protein with sequence similarity to mammalian Insig – InsA (AFUB_064770) is present in the genome but is yet to be characterized in *A. fumigatus*. Moreover, it is plausible that sterol levels fluctuate with filamentous fungal development in batch culture perhaps explaining the apparent constitutive cleavage of *A. fumigatus* SrbA in sterol replete conditions. Intriguingly, mRNA levels of SrbA do not change in the presence of voriconazole in at least 2 independent studies (da Silva Ferreira et al., 2006; Krishnan et al., 2013). This is noteworthy as SrbA has been observed to bind its own promoter in response to hypoxia and azole drug treatment (Chung et al., 2014; Gsaller et al., 2016). Thus, it is also plausible that SrbA activity in *A. fumigatus* is regulated by another signal related to oxygen and antifungal drug responses. Given that genes encoding key enzymes in heme biosynthesis are transcriptionally regulated by SrbA, heme or heme intermediates are a potential possibility through which SrbA activity is regulated that remains to be explored (Davies and Rine, 2006; Chung et al., 2014).

An additional important mechanistic difference in regulation of SREBP activity in *S. pombe* and *A. fumigatus* compared to mammalian cells is at the level of proteolytic cleavage. *S. pombe* and *A. fumigatus* SREBP pathways lack homologs of S1p and S2p. Sre1 is activated by a different mechanism requiring golgi resident defective in *sre1* cleavage (Dsc) proteins which form a complex collectively referred to as the Dsc complex that encode components of an E3 ligase (Stewart et al., 2011). Homologs of *S. pombe* Dsc1-4 are present in the *A. fumigatus* genome (*dscA-D*) and genetic null mutants largely phenocopy the *A. fumigatus srbA* genetic null mutant including important azole drug and virulence phenotypes (Willger et al., 2012). For example, loss of DscA significantly reduces SrbA N terminus protein levels in normoxia and hypoxia and mRNA levels of known SrbA target genes including *cyp51A.* Phenotypes of Δ*dscA* could largely be rescued by ectopic expression of the SrbA N terminus, amino acid residues 1-425. These data link Dsc proteins with regulation of SrbA function in *A. fumigatus.*

Recently, a homolog of *S. pombe* Dsc5, another component of the Dsc complex, was characterized (*dscE)* in *A. nidulans* and showed to be essential for low oxygen survival in this model organism. *A. nidulans dscE* has a UAS and UBX domain at the C terminus, similar to *S. pombe* Dsc5 (Stewart et al., 2012; Bat-Ochir et al., 2016). Interestingly, non-sense mutation in UAS domain of *dscE* results in loss of function of this key hypoxia regulator indicating a role of the UAS and possibly downstream UBX domain of DscE in hypoxia fitness, similar to the mechanism of Sre1 cleavage in *S. pombe* (Bat-Ochir et al., 2016). The UBX domain is known to interact with Cdc48, a major regulator of the Endoplasmic Reticulum Associated Degradation (ERAD) pathway (reviewed in Stolz et al., 2011). In *S. pombe*, the UBX domain of Dsc5 is essential for recruitment of Cdc48 to Dsc2, a component of the Dsc complex; however, this recruitment is intriguingly dispensable for Sre1 cleavage (Stewart et al., 2012). It is worth noting that loss of Cdc48 is indispensable for Sre1 cleavage in *S. pombe*. Thus, further studies are needed to confirm if Cdc48 is recruited to other components of the Dsc complex apart from Dsc2 in a Dsc5-UBX domain independent manner or if expression and not recruitment is essential for its function in Sre1 cleavage. At this time, the role of the UAS domain of Dsc5/DscE is not completely understood. Future studies will determine if the UAS domain has a role in recruitment of Cdc48 or association of Dsc5 to other components of Dsc complex.

**FIGURE 2:**
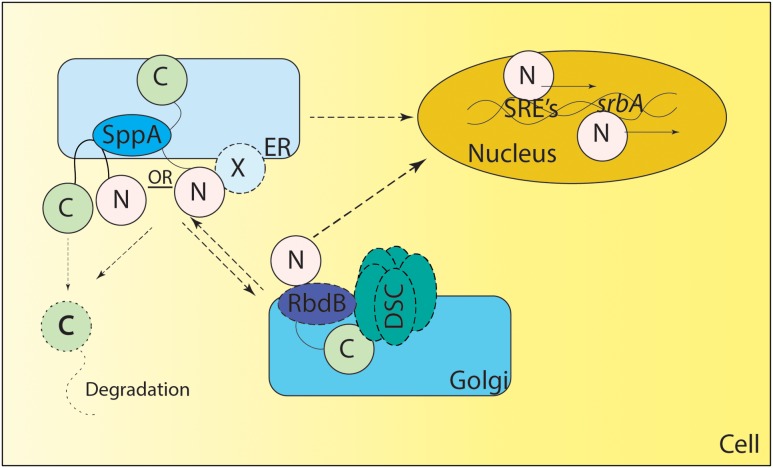
**Proposed Model of SREBP regulation in *A. fumigatus*.** SrbA (full length SrbA protein is represented as N terminus and C terminus joined by a transmembrane region) is an ER resident protein, however, the membrane topology of SrbA is unknown. Unlike *Schizosaccharomyces pombe*, SCAP has not been identified in *A. fumigatus*, and it is not clear if an unidentified ER resident protein “X” forms a complex with SrbA to regulate SrbA activation. Golgi resident Dsc proteins (DscA-E collectively known as DSC complex) are indispensable for SrbA cleavage and activation, however, it is unclear if there is anterograde and/or retrograde movement of SrbA to the Golgi or Dsc complex movement to the ER for SrbA cleavage. The rhomboid protease RbdB is indispensable for *A. fumigatus* cleavage and in *S. pombe* Rbd2 interacts with the UBX domain of Dsc5 (DscE homolog of *A. fumigatus*) via Cdc48. However, the nature of this interaction needs validation in *A. fumigatus*. The Signal Peptide Peptidase (SppA) is an ER resident aspartyl protease involved in regulated intramembrane proteolysis and is indispensable for SrbA cleavage, however, it is not clear if SppA cleavage is preceded or followed by action of the Dsc complex and/or RbdB mediated cleavage. Once cleaved, the C terminus of the protein is potentially degraded and the N terminus translocates to the nucleus where it binds to SRE elements in the promoter region of genes involved in the hypoxia response. SrbA also positively regulates its own mRNA levels by binding the SRE element in the promoter region of *srbA*. Solid lines depict experimentally validated results, whereas dotted lines indicate predicted but not experimentally tested mechanisms. X – Unknown ER resident protein or unidentified SCAP (like) homolog.

Consequently, the mechanism of Sre1 and SrbA cleavage remains to be fully elucidated. Recently, a rhomboid protease Rbd2 has been suggested to be necessary for cleavage and activation of Sre1 in *S. pombe* (Kim et al., 2015). Further mechanistic studies are necessary to determine the exact role of Rbd2 and other yet unidentified mechanisms for cleavage and activation of Sre1 in fission yeast. Recently two proteases including an Rbd2 homolog – *rbdB* and *sppA* - have been identified in *Neurospora crassa* and *A. fumigatus* mediating the cleavage of SrbA (**Figure 2**) (Bat-Ochir et al., 2016; Dhingra et al., 2016; Vaknin et al., 2016). How SrbA interacts with the Dsc complex, RbdB and SppA is an important area of ongoing research. It is important to note that canonically both rhomboid protease and aspartyl protease cleave the intramembrane domains and/or near the N terminal site of their substrates through regulated intramembrane proteolysis (Lohi et al., 2004; Ha et al., 2013; reviewed in Sun et al., 2016). In *A. fumigatus* both proteases appear essential for SrbA activation, thus future experiments will reveal if their functions are conserved or if they act in a non-canonical fashion. It is important to note that the DscE3 complex and SppA are golgi and ER resident localized proteins respectively (Stewart et al., 2011; Willger et al., 2012; Bat-Ochir et al., 2016). Thus, it is unclear if the processing of nascent SrbA happens in ER or if it is a dynamic process requiring anterograde and retrograde transport from ER to golgi and vice versa (**Figure 2**). Additionally, in mammals, SREBP cleavage in the ER lumen by S1P is a prerequisite for S2P activity, thus additional, yet unidentified, proteases may be involved in activation of SrbA (reviewed in Sun et al., 2016). As mammals and *A. fumigatus* have a distinct mechanism of SREBP activation, molecular dissection of SrbA proteolytic cleavage in *A. fumigatus* is an important area of future investigation to fully understand sterol biosynthesis and harness this pathway for novel therapeutic development.

Regulation of SREBPs in other fungi is complex and involves additional regulatory layers including post-translational mechanisms that could potentially be unique to filamentous fungi. These mechanisms are high priority targets for investigation in *A. fumigatus.* For example, a second non-sterol dependent pathway also controls levels of Sre1 in *S. pombe*. When oxygen concentration is high, a prolyl 4-hydroxylase-like 2-oxoglutarate-Fe(II) dioxygenase Ofd1 negatively regulates Sre1N levels in a proteasome dependent manner (Hughes and Espenshade, 2008). Under low oxygen concentrations another protein, Negative Regulator of Ofd1 – Nro1, binds to the C terminal degradation domain of Ofd1 and prevents Sre1N degradation leading to stability and accumulation of Sre1N in cells (Lee et al., 2009). It is interesting to note that while a putative homolog of Ofd1 is present in the *A. fumigatus* genome and remains to be characterized, a homolog of Nro1 is not present bringing into question whether this elegant regulatory mechanism is in play for SrbA regulation.

In mammals, SREBP TF levels and function are also controlled by post-translational modifications and this remains a promising area of investigation in *A. fumigatus.* In mammalian cells, GSK-3 (glycogen-synthase kinase) phosphorylates SREBP1 in response to DNA binding. GSK-3 mediated phosphorylation leads to docking of the ubiquitin ligase FBW7 on phosphorylated residues and subsequent proteasome-mediated degradation of active SREBP TFs (Sundqvist et al., 2005; Punga et al., 2006; Bengoechea-Alonso and Ericsson, 2009). Thus, in *A. fumigatus*, levels of active SrbA may be regulated through phosphorylation by GSK-3 and/or a different kinase followed by proteasome mediated degradation. This hypothesis awaits testing though is particularly attractive given the plethora of kinase inhibitors available to explore for antifungal development.

Another important area for investigation is the relationship between multiple SREBP genes in *A. fumigatus* and sterol biosynthesis. Similar to the mammalian SREBP pathway, three SREBPs have been identified in *A. fumigatus* – SrbA, SrbB, and SrbC (Willger et al., 2008; Chung et al., 2014). A second SREBP, Sre2, is present in *S. pombe*. Sre2 contains the hallmark tyrosine residue in the bHLH DNA binding domain, and two transmembrane domains however, Sre2 does not bind Scp1 and is not regulated by levels of sterols. Interestingly, Sre2 is constitutively cleaved and requires the Dsc complex for processing (Hughes et al., 2005; Stewart et al., 2011). However, loss of Sre2 does not appear to affect hypoxia fitness in *S. pombe.* Further studies are needed to determine the role and targets of Sre2 in *S. pombe*.

In *A. fumigatus*, SrbB and SrbC both have the canonical tyrosine residue in the bHLH DNA binding domain but lack the predicted transmembrane and C terminal domains of SrbA. SrbB mRNA levels are massively induced in response to hypoxia through an unknown mechanism. SrbA contributes in part to *srbB* hypoxia mRNA levels through direct binding to the *srbB* promoter region (Chung et al., 2014). However, loss of SrbA does not completely attenuate *srbB* mRNA levels. Importantly, loss of SrbB in *A. fumigatus* attenuates low oxygen fitness and virulence but not tolerance to azole antifungal drugs. Direct target genes of SrbB remain to be elucidated, but initial studies suggest it contributes to regulation of *erg25A* and *hem13* mRNA levels. Importantly, SrbB is a critical regulator of the ethanol fermentation pathway through direct regulation of the alcohol dehydrogenase *alcC* mRNA levels (Grahl et al., 2011; Chung et al., 2014). SrbC is expressed at low levels in conditions examined to date including low oxygen and its role in sterol biosynthesis and SREBP gene regulation are under investigation (Chung et al., 2014).

An important area of future research is determining how the 3 SREBPs coordinate target gene expression which is expected to yield novel insights into the role of these TFs in *A. fumigatus* biology and pathogenesis. There at least appears to be co-regulation of the key ergosterol biosynthetic genes *erg1* and *erg25A* by SrbA and SrbB (Chung et al., 2014). In mammals, hetero-dimerization of SREBPs controls regulation of SRBEP target genes (Datta and Osborne, 2005). Whether this co-regulation of sterol biosynthesis genes observed in *A. fumigatus* involves heterodimer formation between SrbA and SrbB is not known, and direct regulation of SrbB by SrbA and vice-versa cannot be ruled out and is likely. It is interesting to note the presence of a canonical SRE in the promoter region of SrbA and data suggest SrbA auto-regulates its mRNA levels (Chung et al., 2014). Further research will elucidate if binding of different SREBPs to the SRE element in the promoter region of SrbA confer differential protein regulation for activation of SrbA under hypoxic and sterol-level mediated stress. In general, rigorous promoter analysis of critical genes in ergosterol biosynthesis remains an important but understudied area of *A. fumigatus* biology.

### Regulation of Cytochrome P450 Enzymes

Cytochrome P450 enzymes are heme dependent monooxyge nases and represent an important class of enzymes for normal levels of ergosterol production (and some enzymes in this class are the major targets of the azole class of anti-fungal drugs as previously discussed (reviewed in Nebert and Russell, 2002)). Two major cytochrome P450 heme containing enzymes are present in the ergosterol biosynthesis pathway viz., Erg5 and Erg11 (Cyp51) (Song et al., 2016). Despite their importance in sterol synthesis and removal of toxic sterol intermediates, cytochrome b5 and P450 oxidoreductase are the only known proteins that interact with and control the levels of cytochrome P450 enzymes (Hughes et al., 2007b; reviewed in Pandey and Flück, 2013; reviewed in Schenkman and Jansson, 2003). In fission yeast, the damage response protein Dap1 physically interacts with both Erg5 and Erg11 and is necessary for normal sterol levels (Hughes et al., 2007b). This physical interaction requires heme binding and a stable complex formation between Dap1 and P450 enzymes. Mutant cells lacking Dap1 accumulate sterol intermediates and lower levels of ergosterol (Hughes et al., 2007b).

In *A. fumigatus*, sequence similarity searches led to the identification and characterization of three Dap proteins, DapA-C with antagonistic functions. A DapA null mutant is hypersensitive to itraconazole, whereas a DapC null mutant is more resistant compared to the wild type strain. This sensitivity may stem from the fact that abnormal levels of sterols accumulate in Dap genetic null mutants. A DapA null mutant accumulates lower ergosterol levels and a subsequent increase in levels of ergosta-5,7,24(28)- trienol and ergosta 5,7 dienol indicating a blockage at Erg5 (cytochrome p450 desaturase) (**Figure 1**). The current model of Dap protein mediated regulation of sterol biosynthesis suggests DapA is necessary for stability of Erg5 and Cyp51A and loss of DapA leads to degradation of Cyp51B. Heme binding may be critical for DapA mediated stability of Erg5 and Cyp51A but not Cyp51B. Where DapA is necessary for function of P450 enzymes, DapB and DapC have antagonistic role through iron binding. DapB and DapC are predicted to irreversibly bind iron affecting the local iron concentration and thereby altering heme-dependent P450 enzyme function (Song et al., 2016). The presence of multiple Dap proteins with antagonistic functions allows complex regulation between sterol synthesis and iron availability. As Dap proteins control levels of P450 enzymes, targeting DapA might provide a novel therapeutic target to treat azole resistant cases of IA and or potentiate the efficacy of existing triazoles targeting Cyp51 enzymes.

### ER Stress and Sterol Levels

Stress conditions can overwhelm ER capacity to correctly fold proteins that are destined for the membrane and cytosol. Perturbation in lipid synthesis is known to activate the Unfolded Protein Response (UPR) in *S. cerevisiae* and *A. fumigatus* (Pineau et al., 2009; Han et al., 2010; reviewed in Volmer and Ron, 2015). The UPR pathway alleviates ER stress by balancing the proteins entering the ER for folding and the rate of ER protein folding capacity (reviewed in Moore and Hollien, 2012). Proteins that fail to fold correctly are degraded by a proteasome-mediated degradation pathway called ER-associated degradation (ERAD) (reviewed in Tsai and Weissman, 2010; reviewed in Wang and Kaufman, 2014). Mutants defective in ERAD constitutively activate the UPR, indicating regulatory crosstalk between these two pathways (Travers et al., 2000). *A. fumigatus* detects ER stress via the ER stress sensor IreA, which activates HacA – the major regulator of the UPR (Feng et al., 2011). Ergosterol levels are negatively affected in both *ireA* and *hacA* null mutants and sterol intermediates accumulate in Δ*hrdA*, a strain with a deficient ERAD pathway (Feng et al., 2011; Krishnan et al., 2013). This increase in sterol intermediates in Δ*hrdA* might be the result of HMG CoA reductase turnover as Hrd1 is required for degradation of HMG CoA reductase in yeast (Hampton et al., 1996). Interestingly, Δ*hrdA* is resistant to voriconazole compared to the WT strain, although mRNA levels of *cyp51A* and *cyp51B* are not different in these two strains (Krishnan et al., 2013). While the ERAD pathway appears dispensable for virulence in *A. fumigatus*, mutants that lack the UPR regulators *ireA* and *hacA* show significant virulence attenuation in murine models of IPA (Richie et al., 2009; Feng et al., 2011; Krishnan et al., 2013).

Intriguingly, like the SREBP pathway, the UPR is involved in hypoxia and iron stress responses in *A. fumigatus* (Feng et al., 2011; reviewed in Grahl et al., 2012; reviewed in Krishnan and Askew, 2014). This raises an interesting question of interplay between the UPR and SREBP activation in *A. fumigatus* given also that SrbA is an ER resident protein. It is not clear if SREBP activation requires the UPR, as hypoxia is known to activate UPR in tumors (Feldman et al., 2005; reviewed in Romero-Ramirez et al., 2004; Koumenis and Wouters, 2006). In support of this hypothesis, a mechanism by which the UPR is activated in response to lipid stress is different compared to protein misfolding activation, and *ireA* mediated hypoxia fitness is *hacA* independent in *A. fumigatus* (Feng et al., 2011; Lajoie et al., 2012). As UPR mutants Δ*hacA* and Δ*ireA* have increased sensitivity to azoles and reduced total ergosterol content (Richie et al., 2009; Feng et al., 2011), future research should elucidate whether the role of UPR in mediating azole sensitivity is SREBP dependent or independent. Also, the role of the UPR pathway in azole resistance needs further exploration. This provides a novel genetic opportunity to decipher the link between the UPR, SREBPs and lipid homeostasis and highlights a potential therapeutic opportunity to target the UPR and/or SREBP pathway in *A. fumigatus*.

### Iron Levels Regulate Ergosterol Levels in *A. fumigatus*

Iron is a major cofactor for essential life processes and is indispensable for growth of microbes including *A. fumigatus* (reviewed in Chung et al., 2012; reviewed in Haas, 2012; reviewed in Kaplan and Kaplan, 2009). Microbes have elegant strategies to acquire iron under iron deplete conditions such as in the tissue microenvironments of the host (Schrettl et al., 2007, 2008). Under these conditions, *A. fumigatus* relies mainly on siderophore production for iron acquisition (Schrettl et al., 2004). SrbA is required for production of siderophores and fitness in low iron liquid environments (Blatzer et al., 2011). There is a direct correlation between iron availability and sterol levels in *A. fumigatus* and this is mediated in part by SrbA (Blatzer et al., 2011; Yasmin et al., 2012). Mevalonate is a key metabolic intermediate between ergosterol and siderophore production, and its fate is dependent on iron availability. Under iron replete conditions, mevalonate is preferentially converted to ergosterol, however, in iron deplete conditions mevalonate is converted to the siderophore TAFC. In iron deplete conditions, ergosterol levels are reduced by ∼50% and sterol intermediates accumulate rendering *A. fumigatus* more sensitive to voriconazole (Yasmin et al., 2012). Consequently, these data suggest that HMG CoA reductase is a potential target for treatment of IA. Cholesterol lowering agents known as statins target HMG CoA reductase (reviewed in Tobert, 2003). Statins have fungicidal activity against *A. fumigatus*, however at concentrations higher than safe physiological levels used to control cholesterol in humans (Qiao et al., 2007). In plants, bacteria and protozoa, a non-mevalonate pathway (NMP) exists for isoprenoid biosynthesis (reviewed in Hunter, 2007; Odom, 2011). As NMP is not reported in *A. fumigatus* and homologs of enzymes in NMP are absent in *A. fumigatus*, modifying current statins or development of new drugs to target mevalonate production at clinically relevant concentrations needs further exploration.

## Azole Drug Resistance in *A. fumigatus*

**FIGURE 3:**
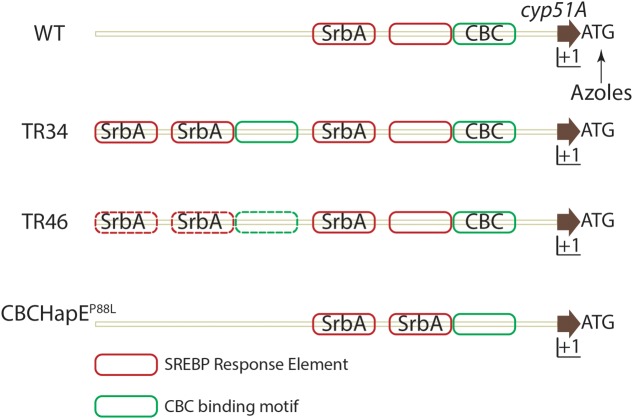
**Regulation of *cyp51A* expression in *A. fumigatus*.** In azole susceptible isolates, two SRE elements and one CBC binding motif is present in the promoter region of *cyp51A*. Binding of SrbA to SREs positively regulates *cyp51A* expression while binding of the CBC to the CGAAT motif negatively regulates expression. Azole challenge (arrow) also positively regulates *cyp51a* expression. In strains containing either a TR34 promoter repeat or TR46 promoter repeat, SRE elements and CBC binding motifs are duplicated and SrbA effectively binds SREs in the duplicated region, thereby increasing *cyp51A* expression. Mutation in the HapE (P88L) subunit of the CBC lowers the affinity of the CBC to the CGAAT motif thereby inhibiting negative regulation of *cyp51A* expression. Solid lines depict experimentally validated results, whereas dotted lines indicate hypotheses which need further validation. Importantly, additional regulatory factors are likely in play at this important gene promoter.

*Aspergillus fumigatus* resistance to azoles was first reported in 1997 (Denning et al., 1997), and is an emerging area of major concern in the fight against aspergillosis (reviewed in Mayr and Lass-Flörl, 2011). Azole resistant isolates have been reported in Europe, Middle east, Asia, Africa, Australia and the USA (Wiederhold et al., 2016). Molecular genotyping has revealed use of azole based fungicides to protect plants as a possible route of azole resistant *A. fumigatus* environmental isolate emergence (Snelders et al., 2009). The dominant resistance mechanism conferring resistance to pan-azole drugs consists of mutations in *cyp51A*, most notably at positions 54, 98, 138, 220 and 448 (reviewed in Denning and Perlin, 2011; Choi et al., 2014). These point mutations may alter the binding affinity of azoles to Cyp51A (reviewed in Parker et al., 2014). Along with mutations in the amino acid encoding region, the presence of tandem repeats (TR) in the untranslated region (UTR) of *cyp51A* also drives an increase in *cyp51A* levels (**Figure 3**) (Snelders et al., 2011; Gsaller et al., 2016). Various tandem repeats have been identified in the UTR of *cyp51* viz., TR_34_, TR_46_ and TR_53_. TR_34_ and TR_46_ are often found in association with mutations in the *cyp51*A gene and the most commonly detected resistance categories to date are TR_34_/L98H, TR_46_/Y121F/T289A (reviewed in Verweij et al., 2016).

A second azole resistance mechanism affecting *cyp51* mRNA levels, exists in *A. fumigatus*. Whole genome sequencing (WGS) of isolates that acquired de novo azole resistance revealed a mutation in the *hapE* gene causing a P88L mutation in the amino acid sequence (Camps et al., 2012). HapE, along with HapB and HapC, is a subunit of the CCAAT binding transcription factor complex (CBC) (Hortschansky et al., 2015). A CGAAT motif is present in the 5′ UTR of *cyp51A* of *A. fumigatus* and the P88L mutation in HapE increases expression of *cyp51A* in *A. fumigatus* (Gsaller et al., 2016).

Recently, an additional understanding of the molecular mechanisms of TR_34_ associated azole resistance was elucidated. In an azole resistant strain with the TR_34_ mutation, the duplication of the tandem repeat (TR) causes duplication of SREs in the UTR of *cyp51A.* SrbA binding to SREs in the TR is responsible for increased mRNA levels of *cyp51A*. However, the CBC, which canonically binds the CCAAT motif, binds a degenerate CGAAT motif in the UTR of *cyp51* and negatively regulates its expression. Consequently, in strains with a mutation in the HapE subunit (HapE^P88L^), CBC binding to the CGAAT motif is significantly reduced allowing increased SrbA SRE binding and increased levels of *cyp51A*. Expression of *cyp51A* is further increased when the TR duplicate is present increasing SRE motifs in the *cyp51A* UTR region (**Figure 3**). Not surprisingly given its role in direct regulation of *cyp51A* levels, loss of SrbA in a TR_34_ azole resistant strain reverses the resistance to azoles and thus provides a potential target to increase the efficacy of azoles against resistant strains (Willger et al., 2008; Blosser and Cramer, 2012; Gsaller et al., 2016).

It is not known at this time if regulation of *cyp51A*, a heme-iron containing enzyme, by the CBC is iron dependent. It is fair to speculate that under iron deplete conditions the CBC will down-regulate *cyp51* expression, thereby optimizing levels of ergosterol and siderophore production under iron deplete conditions. As stated above under low iron conditions, formation of TAFC is favored over ergosterol. This provides a complex layer of regulation and cross talk between iron acquisition and sterol biosynthesis pathways in *Aspergillus fumigatus* that is dependent on *hapX*, the CBC, *cyp51*, DAP proteins, and SrbA. Other recently reported azole resistance mechanisms include overexpression of *cyp51A*, point mutations in *cyp51B* and over expression of drug transporters leading to efflux of drugs (reviewed in Parker et al., 2014). The molecular dissection of the TF interplay at the *cyp51A* promoter is an important advance in understanding azole-*Aspergillus* interactions and warrants further in depth investigation into the factors involved in controlling this critical step in ergosterol biosynthesis.

Recently, an elegant approach that utilized the sexual cycle of *A. fumigatus* identified additional novel loci associated with azole drug resistance. Losada et al. (2015) identified mutations in *cyp51A*, multi-drug transporters, HMG-CoA reductase and interestingly *erg25* from an *in vitro* drug selection experiment. In addition, Abdolrasouli et al. (2015) recently used a WGS approach to better understanding azole resistance mechanisms in a panel of 24 isolates collected across geographic locations. Their study further emphasizes the power of WGS to identify potential azole resistance mechanisms and paves the way for robust genome-wide association (GWAS) studies in this important human pathogen.

### Role of Hsp90

Hsp90 is molecular chaperone that regulates diverse client proteins, many of which are involved in cell signaling (Cowen and Lindquist, 2005). Hsp90 is conserved across eukaryotes and gene deletion strategies reveal it is necessary for survival in *A. fumigatus* (Lamoth et al., 2014). In *S. cerevisiae* and *C. albicans*, Hsp90 function is critical for the emergence of azole resistance. Hsp90 mediated azole resistance is associated with loss of *erg3* function, leading to accumulation of ergosta-7,22-dienol in *C. albicans*. This prevents accumulation of toxic 14-α-methyl-3,6-diol. This allows, alternatively, 14-α methyl fecosterol to incorporate into the membrane and circumvent *cyp51* mediated azole activity (Martel et al., 2010; reviewed in Shapiro et al., 2011). Thus, it is plausible that Hsp90 directly or indirectly affects the ergosterol biosynthetic pathway in *A. fumigatus*. Mutagenesis experiments revealed deacetylation of K27 is necessary for Hsp90 mediated azole resistance in *A. fumigatus*, however no positive interaction was observed between a lysine deacetylase inhibitor and azole drugs in *A. fumigatus* (Lamoth et al., 2014). As *erg3* associated azole resistance is reported for *C. albicans* (Kelly et al., 1997) and has not been identified or reported in clinical azole resistance strains of *A. fumigatus* to date, further research is needed to determine the role of Hsp90 on long-term azole therapies for IA and to completely understand the role of Hsp90 in azole mediated drug resistance.

## Future Directions

The combination of the increasing incidence of aspergillosis across multiple diverse patient populations and the emergence of azole drug resistance highlights the need for therapeutic advances. More in depth research is needed on mechanisms of sterol biosynthesis in *A. fumigatus* given its importance in fungal viability and interactions with clinically relevant antifungal drugs. A common theme in the mechanisms of sterol biosynthesis studied to date is the need for oxygen and iron highlighted by the critical role of the hypoxia and sterol biosynthesis transcriptional regulator SrbA and co-regulatory factors such as the CBC. Given the critical role of SrbA in drug tolerance and susceptibility and its virulence profile *in vivo*, a unique opportunity exists to harness this pathway for therapeutic development (Willger et al., 2008).

While many approaches are feasible, one inexpensive and clinically available approach is to test the hypothesis that alleviating tissue hypoxia can improve disease outcomes through alteration of the SrbA pathway *in vivo*. HBO (100% O_2_ at 2.5–3.5ATA) has been used clinically to treat hypoxia and increase oxygen levels in tissues in multiple disease settings (Kurt et al., 2015; Kolpen et al., 2016; reviewed in Thom, 2011). Importantly, multiple lines of evidence and preliminary results suggest synergy between HBO and action of anti-fungal drugs in some disease settings (Garcia-Covarrubias et al., 2002; John et al., 2005; Segal et al., 2007). It is important to note that MIC’s of azoles including voriconazole against *A. fumigatus* were similar under normoxic and hypoxic conditions (Binder et al., 2015). This might be because SREBPs have evolved to sense sterol levels, thus increasing expression of ergosterol biosynthetic genes under hypoxic conditions (Blosser and Cramer, 2012; Chung et al., 2014). It is plausible that HBO can directly inhibit activation of the SrbA pathway *in vivo* in *A. fumigatus* and thereby enhance the efficacy of azole antifungal drugs, overcome SrbA mediated drug resistance due to over expression of *cyp51A* (**Figure 3**) (Gsaller et al., 2016), and mitigate fungal proliferation. Ergosterol can regulate its own levels through negative feedback regulation in *S. cerevisiae* (Casey et al., 1992). Thus, it is possible that under HBO conditions, increased ergosterol levels would inhibit the pathway and increase sensitivity to azoles. Future research should determine the sterol profile under HBO conditions and if differential sterol profiles play a role in the observed synergy between HBO and anti-fungal drugs in some species. It is possible HBO targets an additional pathway which compliments the targeting of ergosterol. HBO has been shown to increase oxygen free radicals and can increase the activity of immune cells against fungal infections (Almzaiel et al., 2013). HBO is also known to increase angiogenesis and play a role in wound healing (reviewed in Moen and Stuhr, 2012). While HBO may or may not prove to be clinically relevant in the context of IPA, similar approaches to alter the induction of genetic networks critical for sterol biosynthesis and azole drug susceptibility is an important and exciting area of future research.

Other potential therapeutic options include prevention of SrbA cleavage and activation through a direct targeting approach. The genetic null mutants of the strains lacking *srbA*, members of the Dsc complex and proteases encoding *rbdB* and *sppA* genes phenocopy the *srbA* null mutant (Willger et al., 2008, 2012; Bat-Ochir et al., 2016; Dhingra et al., 2016). Thus, screening and identification of small molecules that prevent cleavage and activation of SrbA either by binding the regulatory proteins or inhibiting protein-protein interactions may prove to be beneficial in achieving successful outcomes for IPA. To achieve these goals, additional research is needed to provide a deeper understanding of the underlying molecular mechanisms to identify therapeutic opportunities.

## Author Contributions

All authors listed, have made substantial, direct and intellectual contribution to the work, and approved it for publication.

## Conflict of Interest Statement

The authors declare that the research was conducted in the absence of any commercial or financial relationships that could be construed as a potential conflict of interest.
